# An interesting twist: 90 year-old female with acute small bowel obstruction due to midgut volvulus

**DOI:** 10.1093/jscr/rjae001

**Published:** 2024-01-24

**Authors:** Hillary M Jackson, Khaled Saed, Turner Adams, Matthew B Maturasingh, Fallon D Vedros, Mohammad M Masri

**Affiliations:** Department of Graduate Medical Education, Larkin Community Hospital, South Miami, FL 33143, United States; Department of Graduate Medical Education, Larkin Community Hospital, South Miami, FL 33143, United States; Ross University School of Medicine, 2300 SW 145th Ave #200, Miramar, FL 33027, United States; Faculty of Medical Sciences, University of the West Indies, St Augustine, Trinidad and Tobago; Medical University of the Americas, Charlestown, Nevis, West Indies; Department of Graduate Medical Education, Larkin Community Hospital, South Miami, FL 33143, United States

**Keywords:** malrotation, Ladd’s bands, adult malrotation, adult midgut volvulus, small bowel obstruction

## Abstract

We report an exceptionally rare presentation of midgut volvulus secondary to malrotation in a nonagenarian female. According to our extensive literature review, this 90-year-old female is the oldest reported case of midgut volvulus. This patient presented with acute recurrent emesis. Imaging showed midgut volvulus with associated small bowel obstruction. The patient underwent an exploratory laparotomy that revealed midgut volvulus because of congenital malrotation and Ladd’s bands, necessitating a modified Ladd’s procedure. The patient had an uneventful postoperative course. Congenital malrotation with Ladd’s bands was likely asymptomatic throughout this patient’s life. Our case adds to the scarce instances where midgut volvulus with malrotation is identified in elderly patients, underscoring the importance of considering this diagnosis irrespective of age. We recommend including midgut volvulus because of malrotation in a differential list of atypical small bowel obstruction in elderly patients.

## Introduction

Malrotation of the intestine occurs because of an interruption in the normal development of the embryological gut. While this is relatively common in the 1st year of life, it is rarely seen in adults with an estimated prevalence of 0.17% [1, 2]. Peritoneal fibrous bands otherwise known as Ladd’s bands can create a point of potential obstruction in the small bowel. Midgut volvulus is a life-threatening complication of congenital malrotation characterized by the twisting of the intestines around its mesenteric axis. Volvulus of the small bowel can be primary or secondary to other causes including malrotation, postoperative adhesions, tumor, intussusception, or a Ladd’s band as seen in our patient [3].

## Case report

A 90-year-old female with an unknown past surgical history presented to the emergency department from her nursing home with complaints of dark brown emesis. The patient was a poor historian secondary to dementia and her family was unable to provide any further medical history. According to her medical records, she has a past medical history of chronic kidney disease, Alzheimer’s disease, depression, diabetes mellitus, schizoaffective disorder, hypertensive disorder, and insertion of an inferior vena cava filter. Her current medications were unknown.

**Figure 1:**
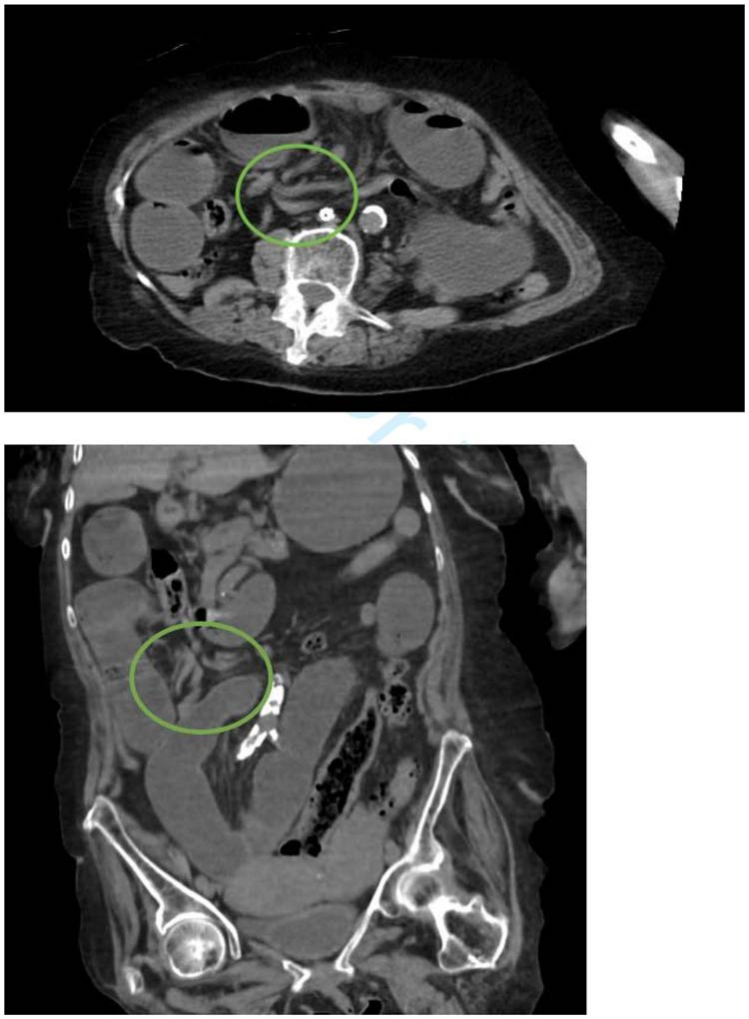
Whirlpool sign indicated by a circle representing a twisting of the mesentery around the superior mesenteric artery.

Initial vital signs included temperature of 98.3°F, respiratory rate of 15 breaths per minute, blood pressure of 140/75 mm of mercury, and pulse of 80 beats per minute. Upon evaluation, the patient was noted to be disoriented. Abdominal exam revealed a benign abdomen without distention, a right paramedian surgical scar, and vertical lower abdominal scar, both well healed. Labs revealed white blood cells of 14.22 white blood cells per microliter and lactate of 2.80 mmol per liter. Computed tomography (CT) showed dilated loops of proximal small bowel with twisting of the superior mesenteric vessels consistent with midgut volvulus (Fig. 1).

**Figure 2:**
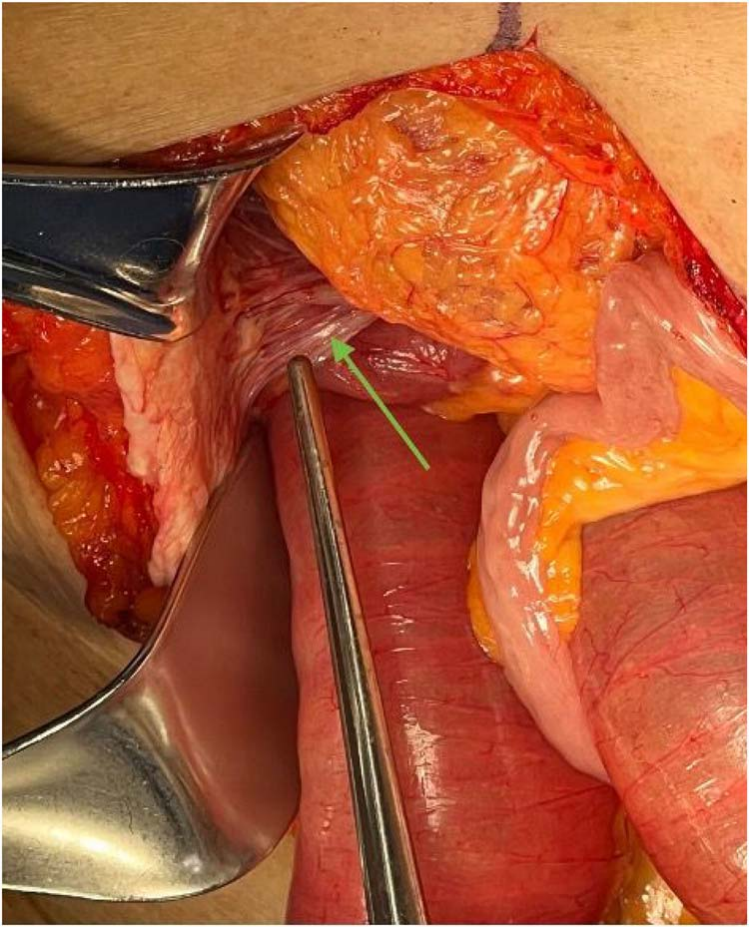
Ladd’s bands are noted in the right upper quadrant during exploratory laparotomy, indicated by an arrow. In this case, these bands adhere the cecum to the abdominal wall.

To attempt decompression, a nasogastric tube was placed and collected 1400 cc of fecal material immediately. The decision was made to take the patient for exploratory laparotomy based on suspected midgut volvulus with associated acute small bowel obstruction. Intraoperatively, the patient was found to have Ladd’s bands and the bowel was found to be twisted around a narrow mesenteric base with the cecum in the right upper quadrant (Fig. 2).

**Figure 3:**
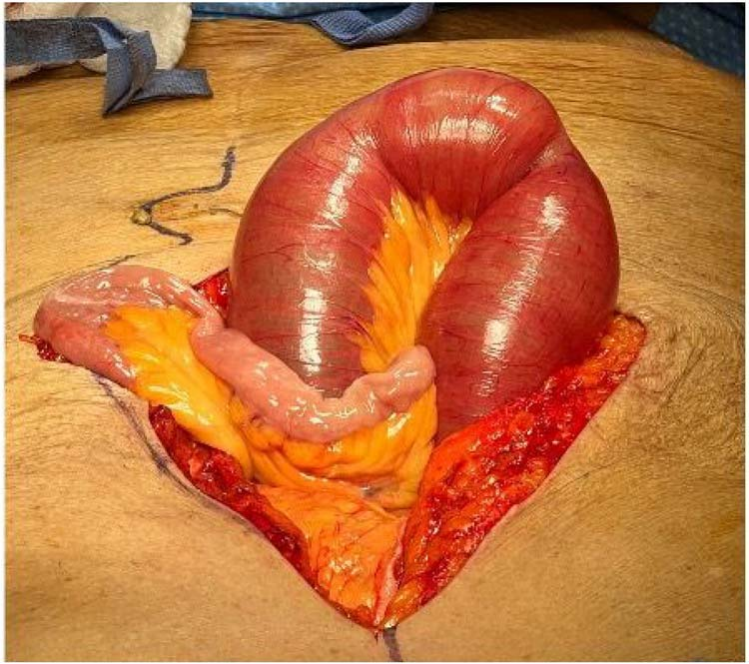
Dilated loops of small bowel proximal to obstruction and collapsed bowel distal to the obstruction.

The small intestines proximal to the midgut volvulus was dilated. The small intestines distal to the volvulus and Ladd's bands was decompressed (Fig. 3).

A modified Ladd’s procedure was then undertaken, foregoing the inversion appendectomy. Postoperatively, there were no complications, and the patient left the hospital on postoperative Day 10.

## Discussion

Midgut volvulus is a condition that is typically encountered in the pediatric population. Midgut malrotation and volvulus could be developed at any age, although it is more common in the first weeks of life. In all, 90% of affected patients will be diagnosed in the first year of life [4]. It refers to the torsion of the small bowel around the superior mesenteric artery [5, 6]. The oldest patient in our extensive literature review discovered to have midgut volvulus because of congenital malrotation with Ladd’s bands was an 82 year old, making our patient the oldest with this pathology [4]. The rarity of this condition in the elderly can create diagnostic confusion and produce dangerous clinical scenarios for already vulnerable patients. Because of its low incidence in adults, diagnosis of midgut volvulus may be overlooked, potentially leading to delayed management and increased morbidity. Symptoms of midgut volvulus may vary between pediatric and adult populations, whereas most pediatric patients present with bilious vomiting in the first month of life, adults can either present with chronic vague abdominal pain or acutely with signs of bowel obstruction and ischemia [7]. A 2020 review of adult presentations of congenital midgut malrotation showed that the symptomatology of midgut volvulus in adults is highly variable with the most common symptoms being intermittent vomiting and abdominal pain [7]. Peritoneal fibrous bands otherwise known as Ladd’s bands can create a point of potential obstruction in the small bowel. Midgut volvulus is a life-threatening complication of congenital malrotation characterized by the twisting of the intestines around its narrow mesenteric base.

A nationwide study in 2016 showed that only 168 cases out of 20 868 cases of intestinal volvulus were because of malrotation as seen in our case [8]. Our patient presented with emesis, without distention or abdominal pain, which is an incomplete clinical presentation of small bowel obstruction. Variable symptoms may complicate diagnosis creating delays in management with subsequent increases in morbidity and mortality with the whirlpool sign only seen in 59% of cases [8]. The Ladd’s procedure, as originally described in 1936 [9], is still the standard treatment of midgut volvulus in pediatric and adult populations.

## Conclusion

After a thorough review of literature, we have found that our 90-year-old patient is the oldest patient to present with malrotation and associated midgut volvulus with small bowel obstruction. Clinical presentation of midgut volvulus in the elderly can be highly variable rendering it an overlooked danger in clinical settings, just as in our case, where the only presentation was acute recurrent emesis with a benign abdomen. Visualization of the “whirlpool” sign on CT is a cardinal sign of midgut volvulus. Increased use of radiologic imaging can help physicians with timely and accurate diagnosis of this rare disorder. A high level of clinical suspicion is essential in navigating this complex diagnosis, enabling prompt intervention in order to reduce morbidity and mortality associated with bowel ischemia, necrosis, and sepsis, which can rapidly escalate in the absence of prompt treatment. It is essential for practitioners to remain vigilant of this condition in their elderly populations as missed or delayed diagnosis can quickly turn deadly.
